# Alkyl Length Effects on the DNA Transport Properties of Cu (II) and Zn(II) Metallovesicles: An* In Vitro* and* In Vivo* Study

**DOI:** 10.1155/2018/2851579

**Published:** 2018-11-11

**Authors:** Itizia Z. Arroyo, Clarissa Gomez, Hugo Alarcon, Araceli Jimenez, Andrew Pardo, Gabriel Montaño, Rodrigo X. Armijos, Juan C. Noveron

**Affiliations:** ^1^Department of Chemistry and Biochemistry, The University of Texas at El Paso, 500 W. University Ave., El Paso, TX 79902, USA; ^2^Center for Integrated Nanotechnologies, P.O. Box 5800, MS 1315, Albuquerque, NM 87185, USA; ^3^School of Public Health, Indiana University Bloomington, Bloomington, IN 47405, USA

## Abstract

Cationic liposomes with DNA-transportation properties have attracted considerable attention for their ability to deliver medicinal oligonucleotides to mammalian cells. Amongst these are metalloliposomes that use transition metal ions to confer the lipid molecules cationic charge and unique advantages such as redox- and ligand-exchange triggered DNA-release properties. In this study, lipophilic copper (II) and zinc (II) complexes of 1-alkyl-1,4,7-triazacyclononane were prepared to investigate their ability to bind and transfect double stranded DNA with mammalian cells in vitro and in vivo. The copper(II)-surfactant complexes Cu(TACN-C8)_2_ (**1**), Cu(TACN-C10)_2_ (**2**), Cu(TACN-C12)_2_ (**3**), Cu(TACN-C14)_2_ (**4**), Cu(TACN-C16)_2_ (**5**), and Cu(TACN-C18)_2_ (**6**) that comprise ligands that vary in the length of the alkyl group and the zinc (II)-surfactant complex of Zn(TACN-C_12_)_2_ (**7**) were synthesized. The critical micelle concentration (CMC) for** 1-7 **was measured using fluorescence spectroscopy and an evaluation of the transfection efficiency of the complexes was assessed using the pEGFP-N1 plasmid and HEK 293-T cells. An inverse relationship between DNA transfection efficiency and CMC of the Cu(II) metallosurfactants was observed. The highest transfection efficiency of 38% was observed for Cu(TACN-C12)_2_ corresponding to the surfactant with dodecyl alkyl chain having a CMC of 50 *μ*M. Further, an* in vivo *experiment using mice models was conducted to test the Cu(TACN-C12)_2_ (**3**) and Zn(TACN-C12)_2_ (**7**) metallosurfactants delivering a DNA vaccine designed for protection against leishmaniasis disease and the study revealed that the Cu-lipoplex elicited the production of significantly more T cells than the Zn-lipoplex and the control group in vivo.

## 1. Introduction

Owing to the improved understanding of biologically active metal complexes, their application in biomedicine has significantly developed in recent years [1]. Considering their cooperative interactions with biological systems, some of these metal complexes are also explored as inorganic therapeutic agents [2]. Copper-based metal complexes are one of the important metal complexes in this class, as copper is present in significant amounts in human brain, heart, and liver [3]. The presence of copper within these vital organs can be understood based on the fact that high metabolic rate of the heart and brain requires relatively large amounts of copper metalloenzymes such as cytochrome c oxidase, dopamine-*β*-hydroxylase, tyrosinase, pyridoxal-requiring monoamine oxidases, and Cu- Zn superoxide dismutase [4]. In recent times, the study of copper complexes for* in vitro *therapeutic applications has gained interest among researchers [2]. For instance, copper complexes were investigated as potential agents to treat neurodegeneration with glyoxal-bis(N(4)-methyl-3-thiosemicarbazonato) copper(II), which was reported to have neuroprotective action in cell culture and animal models of Alzheimer's disease [5]. Similarly, a Cu(II)-picolinate complex was shown to be nontoxic in rat hepatic cells and exerted a hypoglycaemic effect in STZ-induced type 1 diabetic mice [6]. In a different study, it was also shown that the infectivity of influenza A virus was reduced after exposure on copper surfaces [7].

On the other hand, high concentrations of Cu(II) and Zn(II) ions have been implicated in several neurodegenerative diseases that implicate the disruption of the transport and regulation mechanisms of transition metals [8]. Metal transport and permeation into cellular compartments assisted by lipophilic molecules may play important roles in pathology of these diseases. Particularly interesting is the interaction of lipophilic transition metal complexes and oligonucleotides and their ability to cross cellular compartments as indicated by their DNA transfection properties [9–11]. The redox properties and potential for ligand-exchange reactions of transition metals give them wide range flexibility of action during the pathogenesis of disease where they may be implicated [12]. To further understand the biomedical implications that the permeability of metallosurfactants has within biological systems, structure-function studies are necessary. To explore the ability of lipophilic transition metals to bind and transport oligonucleotides in cells, we prepared a series of lipophilic copper and zinc complexes of 1-alkyl- 1,4,7-triazacyclononane with varying alkyl chain lengths and studied their DNA transfection properties in vitro and in vivo. The copper (II)-surfactant complexes Cu(TACN-C_8_)_2_ (**1**), Cu(TACN-C_10_)_2_ (**2**), Cu(TACN-C_12_)_2_ (**3**), Cu(TACN-C_14_)_2_ (**4**), Cu(TACN-C_16_)_2_ (**5**), Cu(TACN-C_18_)_2_ (**6**), and zinc (II)-surfactant complex of Zn(TACN-C_12_)_2_ (**7**) were synthesized. Their critical micelle concentration was correlated to the ability to transfect HEK 293-T cells with the pEGFP-N1 plasmid. Atomic force microscopy (AFM) and optical fluorescence microscopy were used to image the metallo-DNA complex that is able to cross cellular membranes. Further, Cu(TACN-C_12_)_2_ (**3**) and Zn(TACN- C_12_)_2_ (**7**) metallosurfactant complexes were tested using an* in vivo *experiment in mice for increasing the efficiency of a novel DNA vaccine against leishmaniasis disease.

## 2. Experimental Section

### 2.1. Materials

Chemicals used for the synthesis were acquired in of analytical grade and used without further purification. 1-bromooctane, 1-bromodecane, 1-bromododecane, 1-bromobutadecane, 1- bromohexadecane, 1-bromooctadecane, tetrahydrofuran, and sodium hydride were purchased from Fisher Scientific. Cell culture Dulbecco Modified Eagle Medium (DMEM), Fetal Bovine Serum (FBS), Nile Red, SYBR Green, and 1,4,7-triazacyclononane were acquired from Sigma-Aldrich Co. pEGFP-N1 plasmid was a generous gift from Dr. Renato Aguilera at the University of Texas at El Paso. Lipofectamine was purchased from Invitrogen Life technologies. QIAprep Spin Miniprep Kit was used from Qiagen Co. for plasmid extraction. Monitoring of the reaction was performed using 0.25 mm thick Thin Layer Chromatography (TLC) plates. Flash chromatography was performed with SP1 Biotage purification system using 25+M cartridges.

### 2.2. Synthesis of Ligands TACN-C8, TACN-C10, TACN-C12, TACN-C14, TACN-C16, and TACN-C18

The synthesis must be carried in an inert gas like nitrogen. A mixture of 1,4,7- triazacyclononane (300 mg, 2.31 mmol) and sodium hydride (60 mg, 2.55 mmol) in 10 mL of dry THF was stirred for 10 minutes. Upon activation, a THF solution containing the bromoalkane (2.31 mmol) was added dropwise. The reaction mixture was stirred overnight in an oil bath at 60°C. The products were isolated using an automated flash chromatography system (Biotage), using a gradient of chloroform-methanol on a silica column.

### 2.3. Synthesis of 1-Octyl-1,4,7-triazacyclononane (TACN-C8)

In order to purify this CO_2_ sensitive ligand, an acid treatment with nitric acid followed by neutralization with an aqueous solution of KOH was performed. An extraction in chloroform and evaporation of the solvent gave a pale yellow liquid. Yield: 294 mg, 53%. IR bands (compound spread on KBr pellet, cm^−1^) 3426 (N-H), 2920, 2854, 1463, 722 (C-H); ^1^H NMR (CDCl_3_, 300 MHz): *δ* 2.5-3.2 (m, -NH-CH), 0.88 (t, 3H, CH_3_), 1.2-1.5 (m, CH_2_). ^13^C NMR (CDCl_3_, 300 MHz): *δ* 56, 50, 47, 43 (-NH-CH), 31.7, 29.3, 29.1, 27.1-26.6 (m), 22 (CCH_2_-C), 14 (-CH_3_).

### 2.4. 1-Decyl-1,4,7-triazacyclononane (TACN-C10)

After silica column purification a pale yellow viscous liquid was obtained. Yield: 447 mg, 72% IR bands (compound spread on KBr pellet, cm^− 1^): 3419 (N-H), 2924, 2853, 1463, 721 (C-H); ^1^H NMR (CDCl_3_, 300 MHz): *δ* 2.6-3.2 (m, -NH- CH), 0.88 (t, 3H, CH_3_), 1.2-1.5 (m, CH_2_). ^13^C NMR (CDCl_3_, 300 MHz): *δ* 56, 50, 47, 44 (-NH-CH), 31.8, 29.3, 29.5-29.2 (m), 27.2-26.6 (m), 22.6 (C-CH_2_-C), 14 (-CH_3_).

### 2.5. 1-Dodecyl-1,4,7-triazacyclononane (TACN-C12)

The ligand was obtained as a pale yellow viscous liquid that solidify at room temperature into a white solid. Yield: 573 mg, 83%. IR bands (KBr pellet, cm^−1^) 3443 (N-H), 2922, 2852, 1466, 720 (C-H); ^1^H NMR (CDCl_3_, 300 MHz): *δ* 2.5- 3.2 (m, -NH-CH), 2.1 (s, -NH-), 0.87 (t, 3H, CH_3_), 1.2-1.5 (m, CH_2_). ^13^C NMR (CDCl_3_, 300 MHz): *δ* 56.5, 50.3, 46.8, 43.9 (-NH-CH), 31.9, 29.6-29.3 (m), 27.3-26.9 (m), 22.7 (C-CH2-C), 14.1 (- CH_3_).

### 2.6. 1-Tetradecyl-1,4,7-triazacyclononane (TACN-C14)

This ligand was obtained as a pale yellow waxy solid. Yield: 472 mg, 63%. IR bands (KBr pellet, cm^−1^) 3386 (N-H), 2923, 2852, 1465, 720 (C-H); ^1^H NMR (CDCl_3_, 300 MHz): *δ* 2.5-3.2 (m, -NH-CH), 0.88 (t, 3H, CH_3_), 1.2 1.5 (m, CH_2_). ^13^C NMR (CDCl_3_, 300 MHz): *δ* 56.5, 50.3, 47, 43.9 (-NH-CH), 31.9, 29.6-29.3 (m), 27.3-27.2 (m), 22.6 (C-CH_2_-C), 14 (-CH_3_).

### 2.7. 1-Hexadecyl-1,4,7-triazacyclononane (TACN-C16)

552 mg (67%) of this ligand was isolated as a white solid. IR bands (KBr pellet, cm-1) 3386 (N-H), 2918, 2849, 1465, 718 (C-H); ^1^H NMR (CDCl_3_, 300 MHz): *δ* 2.5-2.7 (m, -NH-CH), 2.1 (-NH-) 0.88 (t, 3H, CH_3_), 1.2-1.4 (m, CH_2_). ^13^C NMR (CDCl_3_, 300 MHz): *δ* 57.9, 53.2, 51.5, 46.8 (-NH-CH), 31.9, 29.7-29.3 (m), 28, 27.5-27.3 (m), 22.6 (C-CH_2_-C), 14 (-CH_3_).

### 2.8. 1-Octadecyl-1,4,7-triazacyclononane (TACN-C18)

A pale yellow solid was obtained. Yield: 620 mg, 70%. IR bands (KBr pellet, cm^−1^) 3200 (N-H), 2922, 2852, 1465, 734 (C-H); ^1^H NMR (CDCl_3_, 300 MHz): *δ* 2.5-2.8 (m, -NH-CH), 0.88 (t, 3H, CH_3_), 1.2-1.5 (m, CH_2_). ^13^C NMR (CDCl_3_, 300 MHz): *δ* 56.5, 50.3, 46.8, 43.9 (-NH-CH), 31.9, 29.7-29.3 (m), 27.3-27.2 (m), 22.6 (C-CH_2_-C), 14 (-CH_3_).

### 2.9. Synthesis of Metallosurfactants Cu(TACN-C8)_2_ (**1**), Cu(TACN-C10)_2_ (**2**)_,_ Cu(TACN-C12)_2_ (**3**), Cu(TACN-C14)_2_ (**4**), Cu(TACN-C16)_2_ (**5**), Cu(TACN-C18)_2_ (**6**), and Zn(TACN-C12)_2_ (**7**)

Using an inert gas, a slow addition of two equivalents of the corresponding ligand (2 mmol) was dissolved in acetonitrile and added dropwise into an acetonitrile Cu(OTF)_2_ salt (1 mmol) solution or Zn(OTF)_2_ salt (1 mmol) for 12 h. Upon coordination there is a drastic change in the color intensity of the solution yielding a navy blue solution for the copper complex or yellow solution for zinc complex.

### 2.10. Preparation of Metallosurfactant Liposomes

The cationic metallosurfactants (1 mmol) were dissolved in 100 *μ*L of chloroform followed by the addition of 1 mL of deionized water. The immiscible mixture was sonicated for 15 min or until the sides of the container were clear and did not contain smeared metallosurfactant. The milky emulsion was used with a rotavapor to remove the chloroform and then water was added to compensate for the evaporation and complete 1 mL. The emulsions were prepared under sterile conditions and were used immediately after preparation.

### 2.11. Critical Micelle Concentration (CMC)

In order to determine the minimal concentration required for micelle formation (CMC), a hydrophobic dye, Nile Red, was utilized as fluorescent probe. Measurements of concentrations required for micelle formation in solutions were determined by using the dye and aggregation was measured with the Fluoroskan Ascent F1 fluorometer (Thermo Electron Corp.). Solutions were monitored at 640 nm on a Fluoroskan microplate with an excitation wavelength of 530 nm. The readings integration time was set to be 100 ms. Stock Nile Red solution in water was prepared by diluting 400 *μ*L of a Nile Red solution in acetone (1 mg/mL) to 9.6 mL of water. Sample preparation was carried out by mixing 20 *μ*L of the Nile Red stock solution in water to different concentrations of metallosurfactant for a total volume of 50 *μ*L in each well.

### 2.12. Dynamic Light Scattering (DLS)

The size of the metallosurfactant and lipoplex was measured using the Dynamic Light Scattering (DLS) method. The DLS determinations were performed on a PD2000 DLS Plus (Precision Detectors), with a scattering angle of 90.0°. The data collected was analyzed using the software Precision Deconvolve Version 4.5. The sizes of the particles were acquired in water (with a refractive index of 1.33 and a viscosity of 8.9 x 10^−4^ Pa.s) at room temperature. Quartz cuvettes were washed with diluted acid and heat dried before sample insertion. The hydrodynamic radius of the metallosurfactants and lipoplex were calculated using the automatic mode.

### 2.13. Atomic Force Microscopy (AFM)

Atomic Force Microscopy (AFM) measurements were carried out using the Magnetic Alternating Current mode (MAC) with a Molecular Imaging PicoPlus system. Cantilevers used were type I MAC with a resonant frequency in liquid of 25-35 KHz. An aliquot of prepared metallosurfactant nanoparticle solutions was loaded onto a cleaved mica substrate and allowed to air dry before imaging.

### 2.14. NMR, IR, and UV-Vis Characterization

^1^H NMR spectra were recorded on a Varian Unity 300 or Varian XL-300 instrument. Infrared spectra were recorded using a Thermo Fisher Nicolet (Nexus 470 FT-IR) spectrometer with software package OMNIC v6. Pellets for IR were formed using a hydraulic press at 0.1-3% by weight of the analyte with potassium bromide (Fisher).

Confirmation of metallosurfactant formation was acquired with a Finnigan MAT 95 spectrometer with MAT ICIC II software package. Ultraviolet spectral analysis was conducted with the Perkin Elmer Lambda spectrometer using WinLab software to process the spectra.

### 2.15. DNase I Sensitivity Assay

Briefly, in a typical assay, pCMV-*β*-gal (0.6 *μ*g) was complexed with varying amounts of the metallosurfactants (using the indicated lipid; DNA charge ratios of 0.3:1 to 9:1) in a total volume of 20 *μ*L in DMEM. The DNA pEGFP-N1 plasmid was amplified in* Escherichia coli*, extracted using the QIAprep Spin Miniprep. The purity of the plasmid was checked with 1% agarose gel electrophoresis and the DNA was visualized with ethidium bromide. The concentration was recorded using ultra-violet absorption at 280 nm. Once isolated, the samples were kept at -20°C until further use.

### 2.16. DNA-Liposome (Lipoplex) Formation

Using the pEGFP-N1 plasmid, 500 ng of the purified plasmid in water was incubated with 60 *μ*L of the metallosurfactant emulsion for 30 min at room temperature. Upon completion of the incubation time, the lipoplex solutions were used in the transfection of the HEK 293-T cell line and for the in vivo experiment.

### 2.17. Stability Test of DNA-Lipoplexes

An electrophoresis experiment was carried out using pcDNA 3.1 (5428 bp) to probe the stability of the plasmid DNA when forming lipoplexes with Cu(TACN-C12)_2_. The Cu(TACN-C12)_2_ complex was dissolved in DMSO then further diluted to 500 nM using 40 mM buffer (HEPES). The Cu(TACN-C12)_2_ (100 nM) and pcDNA 3.1 (48.6 nM) were combined with a total volume of 10 mL. The reaction vessels were then placed in a 37°C water bath. The reaction was quenched at the desired time periods with 5 mL of the loading buffer (0.1 M EDTA, 40% (w/v) sucrose; 0.5% (w/v) SDS) and stored at -20°C prior to analysis. The reaction mixtures were loading onto a gel contain 1% agarose and ran for 105 V for 45 minutes in a TAE buffer solution. The ethidium-stained agarose gel was imaged and analyzed for plasmid DNA fragmentation.

### 2.18. Lipoplex Studies by Microscopy

Nonpolar dye, Nile Red, was incubated with 1 mmol of metallosurfactants in chloroform for 12 h. Then, the nanoparticle emulsification procedure previously described was followed. pEGFP-N1 plasmid (500 ng) was incubated for one hour with SYBR green. Upon SYBR green intercalation of DNA the Nile Red loaded nanoparticles were mixed with it and incubated for thirty minutes. 50 *μ*L samples were loaded onto a slide and protected with a coverslip. Microscope filters for a UV lamp were used to observe the fluorescence of Nile Red at 485 nm excitation and 497 nm for SYBR green.

### 2.19. Toxicity Studies

Human leukemia monocyte U-937 cells were grown at 35°C for 60 h in RPM1 medium at 5% CO_2_ and kept healthy. Monocytes were activated with serum to mature the cells and prevent any further growth. Cells were washed through centrifuging at 1,500 rpm and suspending on rich RPM1 medium. Then using a hemocytometer 100,000 cells per well were plated and returned to the incubator. The cells were exposed to different Cu(II) (**3)** and Zn(II) (**7**) metallosurfactant concentrations of 10, 80, 150, 220, 240, and 360 *μ*M in a total volume of 200 *μ*l. Triplicates and controls, one with just cells and the other with the different amounts of water corresponding to the amount of water introduced into the medium by the different concentrations to measure osmotic pressure change impact and the 1 *μ*L thymidine control. The cytotoxicity assay was run using a scintillation counter giving radiation emission of 3H Tymidine in counts per minute. Flow cytometry for toxicity measurements of the carriers was performed with a Coulter Epics XL cytometer (Beckman Coulter, Fullerton, CA) using a EXPO32 software B and collecting 10,000 events. U-937 cells were maintained until 80% confluency was reached in the plates in a CO_2_ incubator. Digital images of cells after transfection were obtained using the inverted Axiovert 200 fluorescent microscope, using the Axiovision 3.0 image analysis software.

### 2.20. DNA Transfection

DNA transfection experiments were performed* in vitro *using HEK 293-T cells. Human embryonic kidney (HEK) 293-T cells (Edge Biosystems, Gaithersburg, MD) were cultured at 37°C in Dulbecco's modified Eagle's medium (DMEM) with 10% FBS and 1% ampicillin in a humidified atmosphere containing 5% CO_2_. Cells were seeded in 24-well plate with a density of 100,000 cells/well one day before the transfection experiment with whole DMEM media. Transfection was conducted when cells were 80% confluent. The DMEM with serum was removed, and the wells were washed with PBS three times. The pEGFP-N1 green fluorescent protein-encoded plasmid (BD Clontech. Palo Alto, CA) was amplified in* Escherichia coli *and purified with Maxipreps kit (Promega. Madison, WI). Additionally, closed circular DNA plasmid was purified using equilibrium centrifugation in CsCl-ethidium bromide gradients. DNA-liposome complexes were produced by combining 1 *μ*g circular plasmid DNA in 50 *μ*L serum free media with a 50 *μ*L media solution containing 0.5, 1, 2, 3, 4, or 8 *μ*L of a 1 mM aqueous solution of the copper complexes, respectively. After 15 min of incubation, 0.4 ml of DMEM without serum was mixed with the complexes and then added to cells. Lipofectin (Invitrogen, Carlsbad, CA) was used as a positive control for transfection. Pure plasmid without delivery agent was also used as control. After overnight incubation, wells were monitored by fluorescent microscopy to differentiate transfected (fluorescent) cells from nontransfected cells. Experiments were done by triplicate. The transfection efficiency was calculated dividing the number of transfected cells over the total number of fluorescent cells counted. At least 500 cells were analyzed.

### 2.21. Murine Model Experiments

Three groups of BALB/c strain of mice with four mice in each were vaccinated with the pVAX and pBT vectors and the metallolipoplexes followed by exposure to the* Leishmania major *parasite. All groups of BALB/c female mice were injected a maximum volume of 100 *μ*l via an intramuscular route in the hind leg quadriceps using a one ml syringe with a 30 gauge, 1/2-inch needle (BD Ultra-Fine needle™). The experimental groups were mice groups** A **(5 mg pVax-*BT-ICAM *+ 5 mg pVax-*ORFF-Amastin *+ 32 ng ML-Cu complex** 3**), mice group** B **(5 mg pVax*-BT-ICAM *+ 5 mg pVax-*ORFF-Amastin *+ 32 ng ML-Zn complex** 7**), and mice group** C **(100 mg pVax-*BT-ICAM-l*/pVax-*ORFF*-*Amastin*). Three weeks after immunization and at the end of 8th week postinfection challenge, the mice per experimental group were sacrificed* via *CO_2_ inhalation. The CD4 or T-helper percentage for both spleen and lymph nodes was determined using a multiparameter flow cytometer. About 1.5 x 10^6^ leukocytes from spleen or lymph node cells were incubated with 10 *μ*g/ml leishmania antigen and 2 *μ*l/ml antibody to CD28 for 2 h at 37°C. Brefeldin A was added at final concentration of 10 *μ*g/ml and cells were incubated for 4 hours. To define T cell phenotype and intracellular cytokine staining, cells were incubated with the viability dye ViViD, followed by staining for, CD4, IFN-*γ*, IL-2, and TNF-*α* using the BD Cytofix/Cytoperm kit.

## 3. Results and Discussion

### 3.1. Synthesis and Characterization

**Figure 1:**
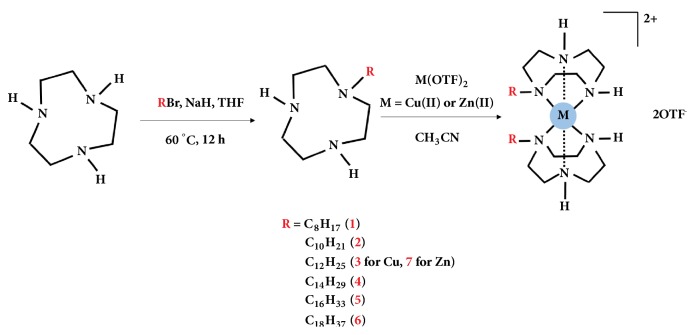
Scheme showing the synthesis methodology for the Cu(II) and Zn(II) metallosurfactants with lipophilic ligands.

The scheme for the generic synthesis of the metallosurfactants is shown in Figure 1. The synthesis of lipophilic ligands was carried* via *the nucleophilic substitution of 1-bromoalkyl compounds with 1,4,7-triazacyclononane (TACN) in THF in the presence of sodium hydride. This was followed by purification with flash chromatography and characterization with ^1^H NMR, ^13^C NMR, and FT-IR, for which the data is given in the experimental section. The corresponding metallosurfactant complexes** 1-7 **were prepared from treating two equivalents of the lipophilic ligands and one equivalent of metal trifluoromethanesulfonate (OTF) salts, Cu(OTF)_2_, or Zn(OTF)_2_, dissolved in acetonitrile. Fractional crystallization using diethyl ether was used to isolate the complexes. The structure of the head group of the metallosurfactants was confirmed using comparative UV-Vis spectra of nonalkylated complexes that were previously characterized with X-ray crystallography [13].

### 3.2. Critical Micelle Concentration

Critical micelle concentration was determined as described in the literature [14]. The lipophilic Nile Red fluorophore was used to track the formation of metallovesicles at varying concentrations.  Table 1 shows the CMC values for the lipophilic complexes** 1**-**7**. The complexes** 2, 3, **and** 7 **showed a CMC value of 50 *μ*M. The complexes** 4 **and** 5 **exhibit a CMC value of 400 *μ*M. The complex** 6** exhibits a CMC value of 800 *μ*M. The metallosurfactant** 1 **did not exhibit fluorescence at any concentration and hence the CMC could not be determined.

### 3.3. Dynamic Light Scattering (DLS)

**Figure 2:**
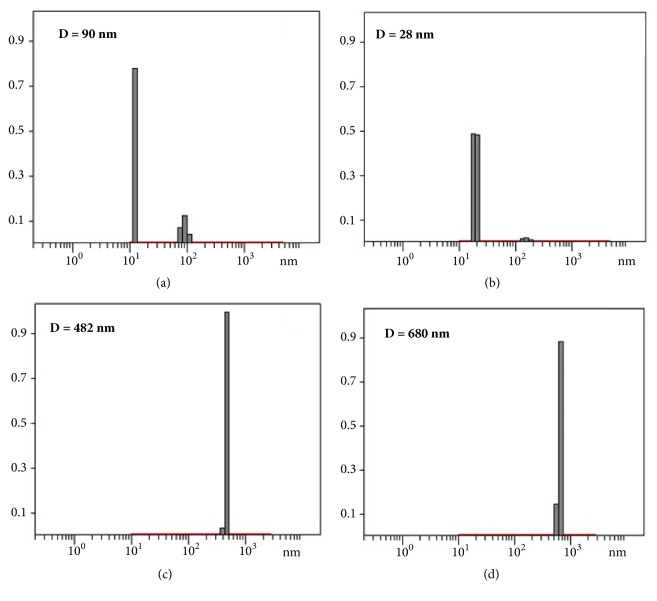
DLS measurements showing the hydrodynamic diameter of (a) Cu(TACN-C12)_2_ (**3**) metallosurfactant in water; (b) Zn(TACN-C12)_2_ (**7**) metallosurfactant in water; (c) (**3)** with pVax; (d) (7) with pVax.

Dynamic light scattering analysis was carried out to determine the hydrodynamic radius of the metallosurfactant particles. Measurements were carried on solutions using 1 mM concentrations. The metallosurfactants** 2**-**7 **yielded a distribution with hydrodynamic radius < 50 nm accounting for 95% of the population. Extrusion procedure using 40 nm filter gave homogeneous dispersions solutions with a particle size centered at 40 nm.  Figure 2 shows the hydrodynamic diameter distribution of the metallosurfactant** 3 **and** 7 **without the plasmid DNA (pVax) and after the plasmid DNA (pVax) complexation.

### 3.4. Atomic Force Microscopy

**Figure 3:**
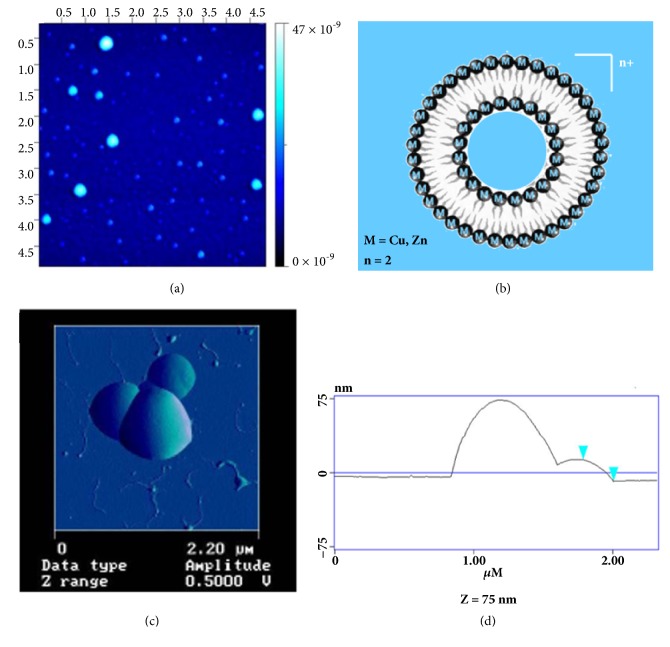
(a) Low-magnification AFM images of the metallosurfactant complex (**3)** on mica surface showing the size distribution ranging from 20 to 50 nm; (b) graphical representation of the metallosurfactant liposome; (c) high-magnification image of the lipoplex formed from the metallosurfactant complexation with DNA; and (d) the height profile of the AFM cantilever showing the size of the lipoplex to be ~75 nm.

Atomic Force Microscopy (AFM) was used to image the vesicles of the metallosurfactants and their interactions with DNA.  Figure 3(a) shows the AFM image of metallovesicles generated from complex** 3 **on a freshly cleaved mica surface. The image shows size distribution of the vesicles ranging from 20 to 50 nm.  Figure 3(b) shows the graphical representation of the metallovesicles with micelle like formation.  Figure 3(c) shows the high- magnification image of the lipoplex formed from the metallosurfactant complexation with DNA and Figure 3(d) shows the size of the lipoplex to be ~75 nm based on the height profile of the AFM cantilever. The DNA plasmid induces particle aggregation and the image reveals the DNA-mediated aggregation of vesicles when they were exposed to the pEGFP-N1.

### 3.5. Microscopy

**Figure 4:**
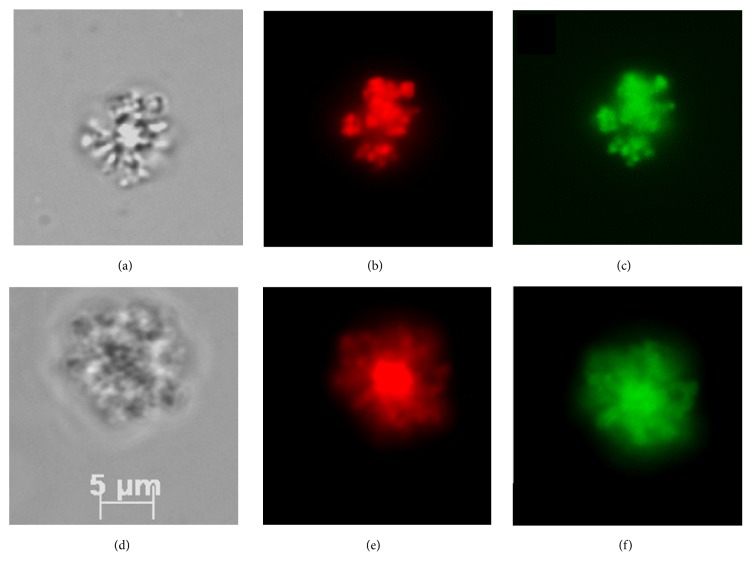
(a) Metallosurfactant complex (**3)** with pEGFP-N1; (b) (**3)** with pEGFP-N1 and Nile Red; and (c)** (3)** with pEGFP-N1 and SBRY; (d) metallosurfactant (**7)** with pEGFP-N1; (e) (**7)** with pEGFP-N1 and Nile Red; and (f) (**7)** with pEGFP-N1 and SBRY.

The lipoplexes formed from the complexation of metallosurfactants (Cu and Zn) and the DNA were studied using fluorescence microscopy (Figure 4). For this study, nonpolar dye, Nile Red was incubated with the metallosurfactants in chloroform and the nanoparticle emulsification procedure was followed. The pEGFP-N1 plasmid DNA was incubated with SYBR green and upon SYBR green intercalation of DNA; the Nile Red loaded metallosurfactants were mixed with it and incubated for thirty minutes. 50 *μ*L samples were loaded onto a slide and protected with a coverslip. Microscope filters for a UV lamp were used to observe the fluorescence of Nile Red at 485 nm excitation and 497 nm for SYBR green. This experiment revealed that the DNA-mediated aggregation of the metallovesicles continues after its initial nucleation at the nanoscale.

### 3.6. DNA Transfection

**Figure 5:**
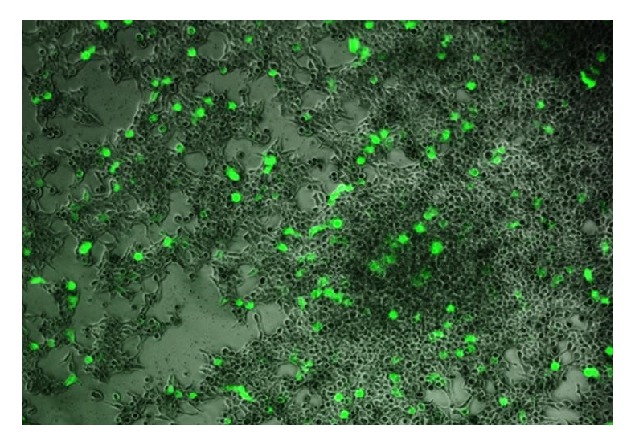
Fluorescence image showing the transfected HEK 293-T cells with the lipoplex formed from pEGFP-N1 and complex** (3).**

The metallosurfactants were complexed with pEGFP-N1 plasmid to form the DNA-liposome complex (lipoplex) which was then utilized for the transfection experiments. Human embryonic kidney (HEK) 293-T cells were used for this* in vitro *transfection process. To observe efficient transfection, cells with a higher density of 100,000 cells/well were used in our experiments. In order to make sure the effectiveness of the metallosurfactants towards the DNA transfection process, control experiments without metallosurfactant (*i.e., *with plasmid only) and lipofectin as a positive control were also carried out. After the completion of the transfection process, the presence of pEGFP-N1 green fluorescent protein-encoded plasmid within the cells was observed under the fluorescence microscope to ascertain the efficiency of transfection (Figure 5). Further, these transfected cells were manually counted to assess the efficiency of individual metallosurfactants. Based on our experiments, we found that the Cu(TACN-C12)_2_ metallosurfactant has an optimal transfection efficiency of 38% that is higher than the efficiency shown by other Cu(II) complexes and the Zn(TACN-C12)_2_ metallosurfactants studied.  Table 1 shows the individual transfection efficiencies of all the metallosurfactants** 1**-**7**.

**Figure 6:**
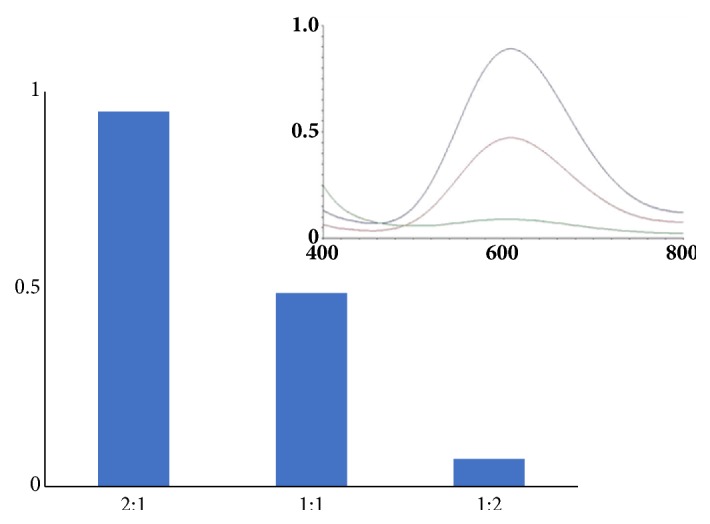
Glutathione-mediated reduction of the complex (**3**) in water.

The Cu-liposomes readily decompose in water in the presence of glutathione (Figure 6), which suggest that this may be a mechanism by which the Cu-liposomes release DNA inside the cells. The Cu(II) ions in the metalloliposomes undergo reduction by one electron with glutathione and result in Cu(I) ions that trigger the decomposition of the amphiphile and the vesicle.

## 4. DNA/Liposome Interaction

### 4.1. Gel Retardation Assay

**Figure 7:**
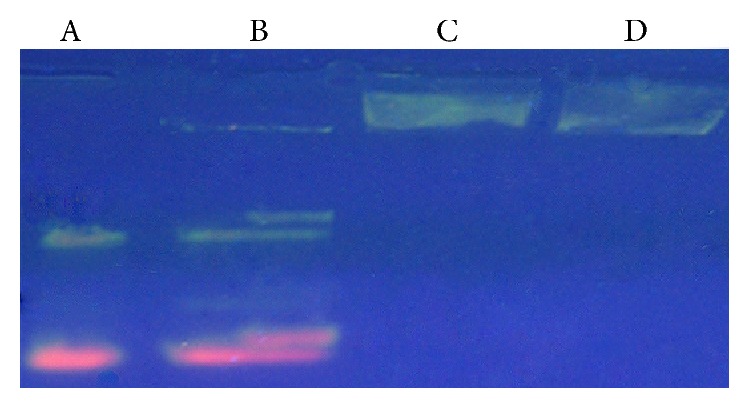
Gel electrophoresis showing the DNA encapsulation by (**3)** and (**7)**. A and B show the individual DNA samples eluted in the gel; C and D show the encapsulated DNA by the metal complexes which are not eluted in the gel.

In order to assay the DNA/liposome ratio to ensure complete encapsulation and thus protection of the vaccine, electrophoresis was chosen to observe interaction between these two complexes (Figure 7).

### 4.2. Ultracentrifugation of Lipoplex

In order to understand the encapsulation efficiency of the metallovesicles with DNA, the bound DNA from the unbound DNA was separated by high speed centrifugation. Taking advantage of the different densities of the lipoplexes, they are precipitated and the supernatant was carefully removed for DNA concentration analysis. In order to reduce error the procedure was done twice. To confirm the results shown by gel retardation analysis this second technique was chosen. The first supernatant did show unbound DNA, but only 3-6% of the original 500 ng used for lipoplex formation. After resuspension of the pellet the second supernatant showed 0-1% unbound DNA. All samples did show an encapsulation efficiency from 99.34-99.97%. This experiment was in accordance with the results found in the gel retardation analysis.

### 4.3. DNA-Lipoplex Stability

**Figure 8:**
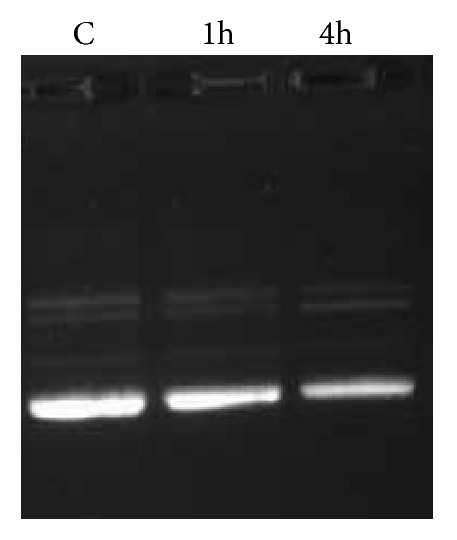
Time-stability of the DNA-lipoplex formed with complex** 3**.

In order to investigate if the Cu(TACN-C12)_2_ complex causes damage onto the encapsulated DNA plasmid, we conducted a time profile experiment under similar conditions where Cu(II) ions are known to cleave DNA [15, 16]. After the DNA-lipoplex was formed using a large plasmid pcDNA3.1 containing 5,428 bp and the Cu(TACN-C12)_2_ complex, it was placed in water at 37°C and the reaction was monitored using gel electrophoresis after 1 and 4 h time intervals. The results showed that there was no indication of DNA cleavage (Figure 8). This is in remarkable contrast to the DNA cleavage observed by substitutionally active Cu(II) complexes bearing only one TACN moiety [15, 16].

### 4.4. Toxicity Studies

The toxicity of metallosurfactant vesicles was tested using activated human leukemia monocyte (U-937) cells before their use in DNA transfection. The cells were suspended in RPM1 medium activated with serum and mixed with different volumes of the 1 mM metallosurfactant solutions to obtain different total concentrations in each well. The plates were placed in incubator for 48 hours, and after that they were incubated for another 24 hours with 1 *μ*L of thymidine in each well. Once this period is concluded, the cells were transferred from each well to a filter paper using a vacuum. Then, the cells were lysed with detergent and exposed the engulfed thymidine. ^3^H thymidine enters through the membrane of live cells, therefore, surviving cells get lysed and thymidine content correlates to surviving number of cells when compared to positive and negative controls. The studies revealed that around 50% of U-937 cells were killed at around 15 *μ*M for the copper metallosurfactant** 3** suspension, and 120 *μ*M for the zinc metallosurfactant** 7 **suspension. Transfection experiments were all conducted under this toxicity threshold for all samples.

### 4.5. DNA Vaccine Containing Plasmid Antigens

The* L. major *cocktail vaccine utilized for the inoculation of mice consists of two DNA plasmids, one encoding for the antigen proteins biopterin transporter (BT) and the intercellular cell adhesion molecules (ICAM), as well as plasmids containing the open reading frame (ORFF), the transmembrane protein Amastin, and PVAX, which is the only plasmid approved by the FDA for human vaccinations due to its unstable properties that allow for its easy removal from the body and minimizes the possibility of chromosomal integration [17–20]. The reason that so many genes were utilized in the vaccine was due to the size of the parasite. As they are large in size, minimal protection would exist from the use of only one gene and in turn would make the parasite more viable and adaptable. If only one molecule was targeted in a parasite, the parasite may still be able to replicate due to the possible redundancies in the parasite's biochemistry. The use of BT, ICAM-l, ORFF, and Amastin Leishmania antigens as a prophylactic DNA vaccine constitute a novel approach for the prevention of cutaneous leishmaniasis (CL) caused by* Leishmania major *using* Leishmania mexicana *antigens. The BT molecule of Leishmania was first described in* Leishmania donovani *by Lemley [19]. Recombinant BT previously was found to confer partial protection when used as vaccine candidate for preventing visceral leishmaniasis in a murine model. This is the first attempt to use BT as a vaccine candidate to prevent CL in a mouse model. To date, the ICAM molecule is the only one of its kind found in Leishmania. ICAM-l is located in the parasite nucleus and on its surface. Previous studies characterized this molecule as a Leishmania ligand, which interacts with receptors on phagocytic cells. Our study is the first to use ICAM-l as a vaccine to prevent CL in a mouse model. The ORFF molecule was first described by Myler [18, 19] who suggested that it had protein coding functions. Tewary has shown that this molecule may also confer partial protection against murine visceral leishmaniasis [20]. Finally, Amastin, which has been identified as potential antigen protein candidate [21], belongs to an extended family of external surface proteins on Leishmania. To date, no attempts have been made to utilize this molecule as a CL vaccine candidate.

### 4.6. Murine Model Experiments

**Figure 9:**
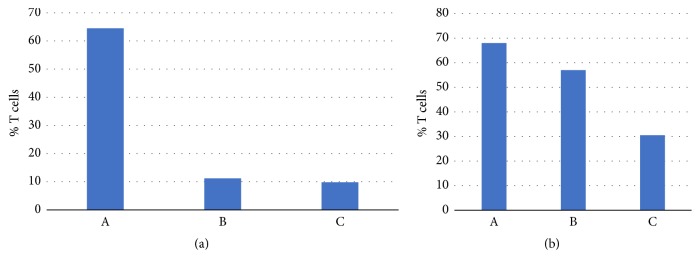
T cell % for (a) lymph nodes and (b) spleen samples in mice group A, group B, and group C.

In order to evaluate the in vivo transfection properties of the Cu- or Zn-metallosurfactants, three groups of the BALB/c strain were vaccinated with the pVAX and pBT vectors and the metallovesicles. Three weeks after immunization and at the end of 8th week after the infection challenge, the mice per experimental group were sacrificed* via *CO_2_ inhalation and the T-helper cells for both the spleen and lymph nodes were quantified since T lymphocytes are a major source of cytokines and bear antigen specific receptors on their cell surface that allow for the recognition of foreign pathogens. The results showed that the Cu-lipoplexes elicited the production of significantly more of T-helper cells relative to the Zn-lipoplexes and the control group (Figure 9). These results indicate that the Cu-lipoplexes are more effective in DNA-delivery in vivo as compared to the Zn-lipoplexes and the control group, which may be due to the increased stability of the Cu-lipoplexes under physiological conditions.

## 5. Conclusions

In conclusion, we report the synthesis of metallosurfactants of Cu(II) and Zn(II) that vary the alkyl length of the lipophilic ligands and correlated with their CMC values and their DNA transport properties in* vitro *and in* vivo*. The metallosurfactants formed lipoplexes with pEGFP-N1 and their DNA transfection efficiency in vitro was analyzed using HEK 293-T-cells. AFM and optical fluorescent microscopy studies indicate that the metalloliposomes form surface-complexes with pDNA that aggregate into clusters in water that nucleate in the nanometer scale and reach sizes in the micron scale. The effect of the alkyl length of the ligands of the metallosurfactants was associated with their CMC values, so that the metallosurfactants with the lowest CMC values of 50 *μ*M exhibited the highest DNA transfection ability of 38% and those with highest CMC exhibited the lowest transfection efficiency. This study established that the thermodynamic stability of the metallovesicles in water, as revealed by the CMC measurements, plays a critical role in the efficiency of DNA transfection. Further, the* in vivo* experiment using mice models to test Cu(TACN-C12)_2_ (**3**) and Zn(TACN-C12)_2_ (**7**) concluded that the Cu-vesicles elicited the production of significantly more T cells at the lymph nodes and spleen samples than the Zn-vesicles and the control groups, which suggests that the Cu-vesicles are possibly more stable than the Zn-vesicles as in vivo carriers of DNA. The use of transition metals in the development of metallovesicles capable of delivering DNA allows for the design of intricate metal-organic structures that respond to physiological conditions that will pave the way to programmable agents for the delivery of oligonucleotide-based medicines in DNA vaccines. In this study, Cu-based amphiphiles clearly demonstrate superior ability to deliver DNA vaccine in murine experimental models.

## Figures and Tables

**Table 1:** Critical micelle concentration (CMC) and transfection efficiency of the metallosurfactants (**1-7**).

Compound	Critical Micelle Concentration (*μ*M)	Transfection Efficiency (Optimal Conditions)
**1**	No micelle formation	0
**2**	50	35
**3**	50	38
**4**	100	25
**5**	400	11
**6**	800	4
**7**	50	35

## Data Availability

All the data here reported to support the findings of this study are included within the article.
