# Corrigendum to “Alkyl Length Effects on the DNA Transport Properties of Cu (II) and Zn(II) Metallovesicles: An *In Vitro* and *In Vivo* Study”

**DOI:** 10.1155/2020/2949201

**Published:** 2020-08-21

**Authors:** Itzia Z. Arroyo, Clarissa Gomez, Hugo Alarcon, Araceli Jimenez, Andrew Pardo, Gabriel Montaño, Rodrigo X. Armijos, Juan C. Noveron

**Affiliations:** ^1^Department of Chemistry and Biochemistry, The University of Texas at El Paso, 500 W. University Ave., El Paso, TX 79902, USA; ^2^Center for Integrated Nanotechnologies, P.O. Box 5800, MS 1315, Albuquerque, NM 87185, USA; ^3^School of Public Health, Indiana University Bloomington, Bloomington, IN 47405, USA

In the article titled “Alkyl Length Effects on the DNA Transport Properties of Cu (II) and Zn(II) Metallovesicles: An In Vitro and In Vivo Study” [1], there was a spelling error in the first name of the first author, listed as “Itizia” instead of “Itzia.” This is corrected as shown above.

## References

[1] Arroyo I. Z., Gomez C., Alarcon H. (2018). Alkyl length effects on the DNA transport properties of Cu (II) and Zn(II) metallovesicles: an *in vitro* and *in vivo* study. *Journal of Drug Delivery*.

